# The Emerging Role of c-Met in Carcinogenesis and Clinical Implications as a Possible Therapeutic Target

**DOI:** 10.1155/2022/5179182

**Published:** 2022-01-13

**Authors:** Antonio Faiella, Ferdinando Riccardi, Giacomo Cartenì, Martina Chiurazzi, Livia Onofrio

**Affiliations:** ^1^Department of Medical Oncology, AO “A. Cardarelli”, Naples, Italy; ^2^Department of Radiology, Oncology and Pathology, Policlinico Umberto I, Sapienza University of Rome, Rome, Italy

## Abstract

**Background:**

c-MET is a receptor tyrosine kinase receptor (RTK) for the hepatocyte growth factor (HGF). The binding of HGF to c-MET regulates several cellular functions: differentiation, proliferation, epithelial cell motility, angiogenesis, and epithelial-mesenchymal transition (EMT). Moreover, it is known to be involved in carcinogenesis. Comprehension of HGF-c-MET signaling pathway might have important clinical consequences allowing to predict prognosis, response to treatment, and survival rates based on its expression and dysregulation. *Discussion*. c-MET represents a useful molecular target for novel engineered drugs. Several clinical trials are underway for various solid tumors and the development of new specific monoclonal antibodies depends on the recent knowledge about the definite c-MET role in each different malignance. Recent clinical trials based on c-MET molecular targets result in good safety profile and represent a promising therapeutic strategy for solid cancers, in monotherapy or in combination with other target drugs.

**Conclusion:**

The list of cell surface receptors crosslinking with the c-MET signaling is constantly growing, highlighting the importance of this pathway for personalized target therapy. Research on the combination of c-MET inhibitors with other drugs will hopefully lead to discovery of new effective treatment options.

## 1. Introduction

MET (Mesenchymal-Epithelial Transition) is a N-methyl-N′-nitro-N-nitrosoguanidine gene aberrantly overexpressed in human osteosarcoma, located in the 7q21-31 loci of chromosome 7. It encodes for a tyrosine kinase receptor and its ligand is the Hepatocyte Growth Factor (HGF). During embryogenesis, MET is essential for several processes including gastrulation, angiogenesis, migration of myoblasts, bone remodeling, and nerve germination [1]. Together with ectodysplasin A, it has been shown to be involved in the differentiation of anatomical placodes, scale precursors, and hair follicles in vertebrates [2]. In adulthood, c-MET was discovered studying epithelial cells which constitutively express it in liver regeneration and wound healing [3]. HGF/MET signaling promotes epithelial cell motility, tissue morphogenesis [4, 5], and mesenchymal-epithelial transition [6]. In fact, its role in pathogenic mechanisms of mesenchyme-derived tumors, such as gastric cancer, and hereditary papillary renal cell carcinoma and also in metastatic head and neck squamous cell carcinomas, childhood hepatocellular carcinoma, ovarian cancer, lung cancer [7–12], and glioma [13–15] is well documented. It seems to be relevant to neutrophil cytotoxicity and its deletion in neutrophils enhances tumor growth and metastatic spread. According to Finisguerra et al. [16], this deletion correlates with reduced neutrophil infiltration in both primary tumor and metastatic sites. Therefore, the efficacy of MET kinase inhibitors in cancer treatment is reduced by the protumoral effect rising from MET blockage in neutrophils.

### 1.1. c-MET/HGF Structure and Pathway

c-MET gene has a total length of 125 kb with 21 exons [17, 18]. Its product is a heterodimer originated from the proteolytic cleavage of a single chain precursor. MET is a glycosylated membrane protein made of a transmembrane *β* chain (145 kDa) and an extracellular *α* chain (50 kDa). The *α* chain heterodimerizes with the aminoterminal extremity of the *β* chain. When Human Growth Factor (HGF) is recognized by c-MET immunoglobulin-like domains and binds the extracellular portion of MET *β* domain, two c-MET heterodimers dimerize, leading to self-phosphorylation of two tyrosine residues within the kinase catalytic domain (Tyr1234, Tyr1235). The heterodimerization leads to the assembling of SEMA domain, the main binding site for the HGF ligand. The extracellular portion of the *β* chain is composed of a cysteine-rich domain, known as the “MET-related sequence” (MRS), and four immunoglobulin-plexin-transcription (IPT) domains. The intracellular portion of the receptor is composed by the catalytic site and a C-terminal tail containing two tyrosines essential for c-MET receptor functions and its oncogenic potential. Ser-985 and Tyr 1003 sites in the juxta-membrane domain play an important role in the negative regulation of c-MET [19, 20]. There are several negative c-MET regulation mechanisms:

- S985 phosphorylation negatively regulates receptorial kinase activity.
- Cbl ligases anchor to the phosphorylated tyrosine Y1003 facilitating receptor ubiquitination and degradation.
- Binding of tyrosine phosphatase (PTPs), including density enhanced phosphatase 1 (Dep1), LAR (leukocyte common-related molecule), PTP1B and T-cell protein tyrosine phosphatase, modulates signal dephosphorylating tyrosines both in the kinase domain and in the docking site [21].
- PLCy activates protein kinase C which decreases phosphorylation and ultimately c-MET activity [22].

HGF, also known as Scatter Factor (SF), plays a role in cells motility by disrupting intracellular junctions, and it is located on chromosome 7q21 [23]. Its sequence contains twenty exons encoding a 92 kDa glycoprotein secreted by mesenchymal cells as an inactive single chain precursor known as pro-HG. Pro-HG is converted into its bioactive forms by cleavage mediated by extracellular proteases. The mature form of HGF consists of a 103 kDa soluble heterodimer made by *α*- and *β*-chain held together by a disulphide bond. The *α*-chain contains an N-terminal hairpin loop followed by four kringle domains (80 amino acid double-looped structures formed by three internal disulphide bonds) and includes a high affinity binding domain for c-MET receptor. The *β*-chain is homologous to serine proteases involved in blood-clotting cascade, but it lacks proteolytic activity [24].

As well known, c-MET can be active by either HGF or its natural isoform NK1. Recently, Uchikawa et al. [25] reported the cryo-EM structures of c-MET/HGF and c-MET/NK1 complexes in the active state. They explained that, throughout two distinct interfaces, one HGF molecule is sufficient to induce a specific dimerization mode useful for c-MET receptor activation. The binding of heparin, as well as the second HGF to the 2 : 1 c-MET : HGF complex, further stabilizes the active conformation. The authors claimed that using cryo-EM and functional analyses, it is possible that the observation of concurrent binding of one ligand HGF to two c-MET receptors, by two completely distinct interfaces, leads to c-MET dimerization and consequently activation. The binding of HGF to c-MET induces the dimerization of c-MET, which enables its intracellular kinase domains to undergo autophosphorylation. In conclusion, the study provides structural insights into the activation mechanisms of c-MET and reveals how two isoforms of the same ligand may activate the c-MET receptor by different mechanisms.

The linkage between c-MET and HGF promotes the autophosphorylation of two intracellular tyrosine-domains (Tyr1349, Tyr1356), like the adapter molecule GRB2-associated-binding protein 1 (GAB1) [26], a scaffolding protein adaptor containing a MET-binding site, which provides binding sites for effectors containing domain Src-homology-2 (SH2), such as the protein of transformation SH2 (SHC), phosphoinositide 3 kinase (PI3K), domain SH2 containing protein tyrosine phosphatase (SHP2), phospholipase C*γ*1 (PLC*γ*1), signal transducer and activator of transcription 3 (STAT3), and RAS GTPase p120 [27–31]. Downstream the phosphorylation, the interaction with Src and SHC [32] is facilitated. The c-MET interaction with the docking molecules [33] activates the mitogen-activated protein kinases (MAPK). SOS-RAS-RAF-MEK-ERK-MAPK pathway promotes both cell survival and proliferation. The MAPK cascade is also responsible for the activation of several targets, including the extracellular response kinases 1 and 2 (ERK 1/2) that migrate from the cytosol to the nucleus where they activate the downstream substrates. Pellicci et al. [34] demonstrated that two main transducer pathways connect phosphorylated c-MET to the MAPK/ERK cascade components: activation of Ras small GTPases following the association of the Grb2-child complex of seven proteins (SOS) to the C-term extremity of c-MET and inhibition of degradation of p120 protein by the interaction c-MET-Gab1-SHP2 [35]. MAPKs also activate the N-terminal Jun kinases (JNK) and p38MAPK. These proteins, in turn, activate cell cycle regulators stimulating cell proliferation and functional changes in the cytoskeleton necessary for cell migration and invasion [36, 37]. Y1356 phosphorylation of c-MET triggers PI3K phosphorylation, which in turn activate focal adhesion kinase (FAK) inducing cell mobility [38]. However, PI3K can be activated by Gab1 leading to inactivation of the BCL2 antagonist of cell death (Bad) and degradation of the proapoptotic protein p53 increasing cell survival [39, 40]. In addition, the phosphorylation of Y1356 residue moves STAT3 to the nucleus where it binds DNA and promotes expression of genes related to angiogenesis and long-term response [41, 42]. Considering its complex role in oncogenesis, c-MET activity is strictly regulated. The main control mechanisms include internalization and degradation/recycling of the receptor [43]. Degradation can occur through various pathways: clathrin-coated vesicle internalization by c-MET binding to the CIN85 and Cbl endophilins [44] and endocytosis independent degradation [45]. Li et al. [46] showed that Cbl is crucial for c-MET degradation and Grb2 is required for c-MET endocytosis. Endosomes could provide a modulating mechanism that allows the receptor to act at the right time and in the right place with the right signaling output [47]. Kermorgant and Parker [48] showed that the translocation of c-MET from the endosomal to the perinuclear compartment depends on PKC*α* and the inhibition of PKC*α* resulted in a reduction of c-MET signaling. They also found in 2004 that PKC regulates both c-MET traffic from early endosomes to perinuclear compartments and c-MET control of ERK in endosomes responsible for the cellular migration [49]. The same group revealed that c-MET signaling is differently regulated when c-MET is localized in peripheral endosomes compared to perinuclear endosomes [49, 50]. Endosomal localization of c-MET is not only meant for degradation, but the receptor can retrotranslocate to the plasma membrane through a process called receptor recycling, which was extensively demonstrated for other RTKs like EGFR. Proteins implicated in plasma membrane receptor recycling include Hrs, Tensin 4, Arf-localized *γ*-ear binding protein-3 containing Golgi (GGA3), and Rab coupling protein (RCP) [51, 52]. A continuous activation of c-MET, determined by increased recycling, leads to a promalignant signaling [53].

### 1.2. c-MET and EGFR

EGFR and c-MET are frequently coexpressed in tumors and these pathways converge on the same downstream signaling mediators such as ERK/MAPK and PI3K/AKT. Crosstalk between EGFR and c-MET in lung cancer has been widely reported [54]. Because of the connection between EGFR pathway and c-MET activation, simultaneous targeting of these two pathways is promising [55]. Several studies highlighted the use of EGFR and c-MET combination treatments, as well as sequential targeting therapies [56–59]. Interestingly, both c-MET and EGFR are known to play a dramatic role in the progression of NSCLC [60, 61]. EGFR inhibitors have been used in several clinical studies [62] alone and in combination with MET inhibitors. Unfortunately, the data obtained from these trials are contradictory. In Neal's study [63], a total of 111 patients were randomized to test the efficacy of cabozantinib vs. erlotinib. In this study, PFS was evaluated which seemed better in the group treated with erlotinib plus cabozantinib. The OAM4558g phase II randomized study evaluated the activity and safety of onartuzumab plus erlotinib vs. placebo plus erlotinib in patients affected by recurrent NSCLC and suggested that only tumors with overexpression of c-MET benefit from the combined treatment [64]. The Asiatic ATTENTION phase III randomized study showed median OS was decreased in patients who received combination therapy of erlotinib with or without tivantinib [65]. The study was stopped prematurely due to an increased incidence of interstitial lung disease in the tivantinib arm. The MARQUEE trial randomized 1048 patients to receive erlotinib with or without tivantinib [66]. This trial was terminated early because of an interim analysis revealing futility and OS did not differ among groups. Recent research has shown that non-small-cell lung carcinomas with acquired resistance to EGFR inhibitors tend to show amplifications in MET [67, 68]. This suggests that combined treatment with EGFR and c-MET inhibitors could be necessary in a subset of patients to avoid the onset of resistance to these drugs. MET amplification is detectable in more than 5% of patients with EGFR mutation-positive NSCLC [66–70].

### 1.3. c-MET and RON

RON (Recepteur d'Origine Nantais) receptor is synthesized as a 185 kDa single chain precursor which is cleaved proteolytically and exposed on cell surface as a heterodimeric glycoprotein including an alpha chain (35 kDa) and a beta chain (150 kDa) [71]. The activities elicited by this receptor family have been termed “invasive growth” [72]. Activation of RON receptor brings the activation of signaling pathways including MAPK and PI3K e *β*-catenin. RON receptor, known as macrophage stimulating 1 receptor (MST1R) or stem cell-derived tyrosine kinase (Stk) in mouse, belongs to the family of tyrosine-kinase receptors. It is made of 1400 amino acids including a signal peptide, an extracellular domain, a single transmembrane domain, and an intracellular tyrosine kinase domain. The extracellular domain and the kinase domain have a 25% and 63% degree of homology respectively with the corresponding domains found in MET receptor. RON expression was found in CNS, kidney, testicles, bones, lungs, and breast and in the GI epithelium. This signaling regulates cytokines and chemokines production in response to inflammatory stimuli [73–79]. RON ligand is the Hepatocyte growth factor-like protein (HGFLP) also known as macrophage stimulating protein 1 (MST1) or macrophage stimulating protein (MSP) [80]. The name HGFLP was given to the first isolated cDNA encoding this protein, based on sequence homology with HGF [81]. HGFLP is mainly secreted by hepatocytes as a biologically inactive single chain precursor. RON and c-MET are coexpressed in many types of cancers, and cross-talking between c-MET and RON has been demonstrated. The interaction of c-MET with RON receptors leads to transphosphorylation of the c-MET receptor in the absence of HGF [82]. It has been recently shown that transactivation of RON by c-MET may be a feature of cancer cells that are “addicted” to c-MET signaling [83]. Recently, transactivation between c-MET and both platelet-derived growth factor receptor (PDGFR) and Axl was found to play a role in bladder cancer [84]. c-MET and RON are structurally related proto-oncogenes belonging to the semaphorin family of transmembrane RTKs [85]. c-MET and RON have both essential functional roles in embryonic development and organogenesis [86] and are overexpressed and/or aberrantly activated in various cancer types suggesting their potential importance as therapeutic targets. They are involved in tumor progression by increasing proliferation, inhibiting apoptosis, and promoting metastasis and maintenance of cancer stem cells [87]. Interestingly, RON has tumorigenic activity by different mechanisms. There are mutations in the promoter related to increased transcription and at least six different RON isoforms in cancer cells that originate from alternative pre-mRNA processing, alternative transcription, or truncation [22].

### 1.4. Small Cell Lung Cancer and Non-Small-Cell Lung Carcinoma

Several comparative studies detected c-MET overexpression in 60% of NSCLC cases. MET amplification and its most relevant somatic splice-site mutation that results in a skipping of exon 14 are both potential predictive biomarkers for non-small-cell lung cancer (NSCLC) [88–93]. The loss of transcription of exon 14, detectable by liquid biopsy or tissue biopsy, occurs in 3-4% of NSCLC and plays a role of primary oncogenic driver [88, 89, 94]. Gene-overexpression is detected by Immunohistochemistry (IHC) in 37–61% of patients; c-MET gene mutations instead are detectable in only 1–6% of cases [88, 94, 95]. Oncogenic mutations were found outside the kinase domain in NSCLC tissue samples, such as mutations in the semaphorin domain (E168D, L229F, S323G, and N375S) and in the juxta-membrane domain (RR988C, T1010I, S1058P, and exon 14 deletions). The phosphorylation of Y1003, located in the juxta-membrane domain, is responsible for internalization of c-MET receptor through interaction with the Casitas B-lineage Lymphoma (CBL) ubiquitin ligase. The loss of Y1003 due to exon 14 deletion seems crucial for c-MET receptor build up on the cell surface contributing to cancer progression [86]. Awad's study showed that MET exon 14 mutation is more frequent in IV stage patients. Moreover, the gene amplification is detectable in 2–5% of NSCLC at first diagnosis and from 5% to 22% in patients with NSCLC with EGFR mutation following erlotinib/gefitinib and more than 20% of patients affected by lung cancer with brain metastases. MET represents a therapeutic target in NSCLC [88–90, 95, 96]. In particular, MEK-inhibitors are tyrosine kinase inhibitors which are divided in nonselective type 1a inhibitors, as crizotinib, and selective type 1b inhibitors, as tepotinib and capmatinib [97–100]. The last two have been studied in two different clinical trials: GEOMETRY and VISION. Wolf et al. have evaluated in the GEOMETRY mono-1 trial, a prospective, international, on-label, multiple-cohort, phase 2 study, safety and efficacy of the selective MET-receptor inhibitor capmatinib in MET-dysregulated advanced/metastatic NSCLC, independently from any histological features and oncogene addiction. They have studied the clinical response and the reduction of measurable lesions studied with imaging techniques in accordance to the Response Evaluation Criteria in Solid Tumors (RECIST) version 1.1. They administered 400 mg twice daily in 364 patients and they observed an Overall Response (Primary Endpoint) in 41% of 68 patients which had previously received one or two lines of therapy and in 68% of 28 patients who had not received treatment before. The median duration of response was 9.7 months among the first group and 12.6 months among the second one; instead, the median progression-free survival was respectively 5.4 and 8.2 months. Capmatinib has demonstrated an intracranial activity; in fact CT scans have shown a disease control in 7 patients and a complete response in 4 patients with brain metastases [100]. In VISION, a multicohort, open-label, phase 2 study, 152 patients affected by advanced NSCLC not-oncogene addicted with MET 14 exon-skipping mutations, have received tepotinib, a high selective oral MEK-inhibitor, at a dose of 500 mg once daily, in order to evaluate its efficacy and side-effect profile. The Objective Response (OR) was the Primary Endpoint and it was reached after 9 months of follow-up. In particular, the partial response, according to RECIST 1.1 criteria, was enriched in approximately half the patients [101]. In SAVANNAH, a multicentre phase Ib single arm study, the investigators are exploring the efficacy of 28-day continuative combined therapy with osimertinib (80 mg oral OD) and savolitinib, a c-MET selective inhibitor (300 mg oral OD or 300 mg oral BID or 600 mg oral OD), in patients with EGFR mutation-positive locally advanced or metastatic NSCLC with MET-driven resistance to osimertinib. The Primary Endpoints are safety and tolerability and assessed for each savolitinib doses. The secondary endpoint is the OR, according the RECIST 1.1 criteria. The trial is ongoing and the estimated date of its completion is 30^th^ September 2022. The combination of a third-generation selective EGFR inhibitor, as Osimertinib, with a selective c-MET inhibitor, as savolitinib, resulted in a good therapeutic strategy with an acceptable tolerability profile [71]. Telisotuzumab vedotin (Teliso-V), an anti-c-Met-directed antibody-drug conjugate, was administered once every 2 or 3 weeks in recent phase I and phase II clinical trials in patients affected by NSCLC. Results in terms of tolerability, overall safety, mDOR, OS, and PFS are encouraging for further clinical development in this setting [102]. In a recent Chinese multicentre, single-arm, open-label, phase 2 study, savolitinib has demonstrated an acceptable tolerability profile and a promising activity, with Objective Response Rate (ORR) of 49.2%, in 70 patients affected by pulmonary sarcomatoid carcinoma and other NSCLC subtypes METex14-positive, treated from November 2016 to August 2020 [103]. For what concerns Small-Cell Lung Cancer (SCLC), the activation of HGF/c-MET pathway leads to increased tumor growth and cell survival. Many patients with SCLC have higher plasma levels of HGF and this finding might be explained by MET gene amplification. Aberrant MET pathway signaling was related to activating mutations involving specific domains of c-MET receptor gene. These mutations are responsible for constitutive activation of MET pathway leading to a more aggressive disease. MET phosphorylation may predict a poor clinical outcome [104]. Taniguchi et al. found activation of c-MET pathway in resistant or relapsed SCLC cell lines, which occurred through increased HGF levels and/or MET copy number gain. Interestingly, inhibition of c-MET caused antitumor effects both in vitro and in vivo models [105]. However, there is no scientific evidence regarding the clinical efficacy of c-met inhibitors in SCLC.

### 1.5. Breast Cancer

HGF and c-MET are frequently coexpressed in invasive breast cancer. Coexpression is often stronger at the infiltrative margins. Moreover, there is a significant correlation with high tumor grade, an increased proliferation index and reduced survival, compared with cancers that are negative for coexpression. A truncating mutation in the deoxyadenosine tract element hyperactivates the HGF promoter leading to the formation of an HGF/c-MET autocrine loop [106]. Mutations in the promoter region of HGF were identified in 15% of European women and in 50% of African American patients. In a study of 130 patients with HER-2-positive breast cancer, both MET and HGF amplification were associated with trastuzumab failure and patients with MET amplified tumors had a shorter time to progression [107]. Ho-Yen et al. [108] showed that the HGF/c-MET pathway is associated with breast cancer progression suggesting that there is a solid rationale for continuing to develop anti-c-MET drugs, particularly for patients without many options available like those with basal-like and triple-negative breast cancer. A wide range of mechanisms can result in aberrant c-MET signaling including activating gene mutations, gene amplification, protein overexpression, increased ligand-dependent paracrine stimulation, and acquisition of autocrine signaling. Ho-Yen et al. found lower c-MET expression in E-Cadherin-negative invasive lobular carcinomas and a higher one in tubular carcinomas (a well-differentiated subtype characterized by angulated tubules) [109, 110]. These observations show reminiscence of findings from studies on mammary development, where HGF stimulated tubule formation in murine mammary epithelial cells [111, 112]. They also demonstrated, for the first time, that c-MET protein expression was independently associated with basal-like breast cancer and its evaluation will be included in clinical trials for anti-c-MET therapy. Instead, MET amplification was considered unusual in this type of malignancy [113, 114].

### 1.6. Kidney Cancer

Germinal mutations in c-MET gene were identified in hereditary and sporadic renal cell carcinoma (RCC). Specifically, c-MET is overexpressed in Von Hippel Lindau Syndrome due to upregulation of hypoxic induced factors which can enhance MET signaling [115]. VHL mutations and loss of heterozygosis for this gene were associated with high c-MET expression in clear cells RCC as well [116]. Kidney cancer with high c-MET expression shows higher nuclear grade (II-IV), pT stage, lymphatic involvement, and poor prognosis. The induction of c-MET activates RAS pathway and prevents apoptosis through cytoprotective enzyme heme-oxygenase-1 (HO) overexpression, which defends cells from killing effects of chemotherapy. Moreover, MET-HO-1 interaction stimulates the synthesis of PD-L1 protein. This protein binds its specific receptor PD-1 expressed by the T cells causing inhibition of cell-mediated immune response against malignant cells. PD-L1 induction triggered by c-MET is involved primarily in cancer immune escape through the PD-1/PD-L1 pathway [117, 118]. This finding pushed Motzer et al. [119] to evaluate the combination of Nivolumab plus ipilimumab versus sunitinib, a well-known c-MET inhibitor, in the setting of advanced RCC. A better PFS was observed in patients treated with nivolumab plus ipilimumab than patients treated with sunitinib. However, these results were achieved in patients with an expression of PD-L1 ≥ 1% and high/intermediate risk. Instead, higher OS and objective response (OR) were found in low-risk patients treated with sunitinib regardless of the level of PD-L1 expression. Considering these findings, prognosis and treatment of patients with metastatic RCC (mRCC) might be based on the molecular classification to define an appropriate target treatment. In addition, antiangiogenic therapy with sunitinib causes hypoxia decreasing the blood supply for cancer cells and this causes an upregulation of c-MET pathway. As a result, the overexpression of c-MET in patients treated with anti-VEGF therapy might explain the reason why patients with higher expression of c-MET have less benefits from a therapy with sunitinib [120]. In CABOSUN randomized phase II trial, the role of cabozantinib, a MET inhibitor which regulates VEGFR and other pathways, was tested in patients affected by mRCC, versus the standard first-line treatment whit sunitinib. According to IMDC criteria, patients were stratified by intermediate (81%) and poor (19%) risk disease classes and also for their MET status. PFS was the primary endpoint and median PFS, according to an independent radiology review committee and to RECIST 1.1 criteria, was 8.6 months for 79 patients who received cabozantinib 60 mg once daily versus 5.3 months in 78 patients who received sunitinib. In addition, median PFS per investigator was respectively 8.3 months versus 5.4. Subgroup analyses of median PFS based on MET level expression revealed a median PFS of 13.8 months in patients with high levels of MET who received cabozantinib, versus 3 months with sunitinib. The secondary endpoints were ORR (20% for cabozantinib versus 9% for sunitinib) and the safety. On the base of CABOSUN study, it is acceptable to consider the efficacy of cabozantinib higher than sunitinib in intermediate/poor risk patients affected by mRCC for its specific target on MET [121].

### 1.7. Head and Neck Cancer

HGF was found to be elevated in head and neck squamous cell carcinoma (HNSCC) patients compared to healthy individuals. Lymph-nodal invasion is a common feature of clinical HNSCC and is predictive of patient mortality. c-MET gene is highly expressed in lymph node metastases in HNSCC [122]. It tends to be present in all stages of metastasis and it is more expressed in patients with N2 and N3 nodal metastasis [123]. Met expression is associated with worse prognosis and lower overall survival [124]. HGF/Met expression levels were found to be negatively related to survival in advanced nasopharyngeal carcinoma [125]. New evidence report that c-Met is overexpressed in the majority of HNSCC cases. 2/3 cases of HNSCC demonstrate phosphorylation in the kinase domain involving Y1235D and Y1230C which play a crucial role as activators of the c-Met pathway [126, 127]. The c-MET-HGF pathway was identified as one of the mechanisms of acquired resistance to epidermal growth factor receptor (EGFR) targeting therapies. EGFR is overexpressed in 90% of HNSCC patients. Acquired EGFR resistance mediated by c-Met activation is a common finding in clinical trials [128, 129].

### 1.8. Gastrointestinal Cancers

A lot of causes of inappropriate MET-activation in gastrointestinal cancers can be identified: amplification and mutation of c-MET gene, with subsequent protein overexpression and kinase activation; transcriptional upregulation from other oncogenes (K-RAS); reduced c-MET receptor degradation; ligand-independent activation; autocrine overexpression of HGF; and environmental conditions such as hypoxia and inflammation. Additionally, RNA silencing of c-MET using lentivirus in gastric cancer cells, leads to suppression of peritoneal dissemination showing a proliferative and metastatic role of c-MET [130]. Downregulation and/or inhibition of c-MET significantly decreased growth, migration, and invasion as well as induced apoptosis of tumor cells in a variety of tumor models [131]. Although genetic mutations of MET gene have been detected in 1-2% of patients with gastroesophageal cancer [132], they are extremely rare in patients with gastric cancer overall. However, Lordick et al. demonstrated that more than 65% of advanced gastric cancers with increased metastatic potential, mainly in the liver, express high levels of c-MET. EGFR phosphorylation in advanced tumors induces HGF-independent c-MET activation by phosphorylation leading to oncogenic activity [133–135].

### 1.9. Hepatocellular Carcinoma

Hepatocellular carcinoma (HCC) is the fifth most common cancer and is the second leading cause of cancer death. HGF/c-MET pathway is activated in about 50% of HCC and the expression levels of these proteins are associated with a poor clinical prognosis. Garcia-Vilas et al. argued that HGF is expressed and released by stellate cells. Data suggest that HGF acts by a paracrine mechanism binding c-MET receptor located on hepatocytes cell membrane [136]. Currently, it seems to play a crucial role for hepatocytes survival mediating the interaction with nearby stromal cells [137]. In addition, in patients with HCC, there is an upregulation of c-MET and/or HGF as it increases cell proliferation [138]. In this setting, c-MET is also induced by factor 1 inducible by hypoxia (HIF-1), and once activated, it can induce the expression of VEGF-A, further improving tumor angiogenesis [139]. Therefore, new therapies are being developed targeting the tumor microenvironment, including endothelial cells, immune cells, fibroblasts, and the extracellular matrix.

### 1.10. Central Nervous System Tumors

HGF/MET interaction is frequently committed in human gliomas and high levels of these molecules correlate with high tumor grade and poor prognosis [13–15, 140, 141]. In primary brain tumors, HGF/MET signaling promotes the downstream activation of different pathways, such as the tyrosine kinase Src, which phosphorylates both FAK (Focal Adhesion Kinase), implicated in cell motility and invasion [140], and PI3K, which activates Akt/PKB in order to promote cell survival. Another one is the Janus Kinase/signal transducer and activator of transcription 3 (JAK/STAT3) signaling which promotes the activation of proliferating factor c-myc, cyclin D1, and the nuclear factor kappa-light-chain-enhancer of activated B cells (NFĸB). The latter promotes the antiapoptotic genes (Bcl-xL, Bcl-2) and it is responsible for uncontrolled nervous cells growth and dissemination in glioblastoma [141]. HGF/MET axis also promotes the Wnt/*β*-catenin and the adaptor protein Sos, which activates the RAS/RAF/ERK/MAPK cascade, and consequently increases cell growth, migration, and metastasis. This process involves astrocytoma and glioblastoma progression [22, 142]. In embryonal central nervous system tumors, like medulloblastoma, MET plays a crucial role in promoting both neoangiogenesis, through the direct activation of vascular endothelial growth factors and cell invasion, through the promotion of matrix metalloproteinases expression [143, 144]. As far as brain metastases are concerned, MET promotes their radioresistance [144]. Chen and Guo [145] studied *in vivo* and *in vitro* glioblastoma model, the role of a MicroRNA (miRNA) called miR-410. They demonstrated by FISH and IHC assay that, as a small non-protein coding RNA, miR-410 negatively modulates MET expression and MET/Akt transduction, because of its binding to the 3′ Untranslated Region (UTR) of MET. As well known, miRNAs can recognize target mRNA by imperfect base pairing to the 3′ UTR and they may induce their degradation or translational repression [146]. Chen's group found that miR-410 inhibits MET expression via specific binding to its 3′UTRs and discovered an inverse correlation between MET and miR-410: if miR-410 is suppressed, MET is highly expressed. Furthermore, in high-grade glioma, MET levels are higher and miR-410 levels are lower, while low-grade gliomas are characterized by higher levels of miR-410 which decreases MET expression. Besides, Chen et al. proved that miR-410 may stop glioma cells proliferation and invasion, through MET-regulated Akt signaling, and also that miR-410 reintroduction in murine xenograft models may suppress MET and reduce tumor survival. Recently, many studies on the potential role of monoclonal antibodies against gliomas have been started. In particular, Rilotumumab, a human IgG2 monoclonal antibody, has latterly completed both phase I and II clinical trials. Even if it has shown an important role in stabilizing disease progression in combination with bevacizumab or temozolomide, on the other hand, it is associated to an increased risk of death [147]. Another mAb, Onartuzumab, which competes with HGF binding [148], has demonstrated inhibition of glioblastoma growth in preclinical studies [149]. However, in phase III trial in lung cancer, it did not show any advantages if combined with bevacizumab [150]. As known, c-MET and VEGFR-2 overexpressions are independently associated to shorter time to progression (TTP) after bevacizumab, a humanized anti-VEGF monoclonal antibody. Carvalho et al. found that GBM have predicted that a concomitant overexpression is associated with the worst OS (13 months vs. 19 months; *p*=0.025), an early resistance and a worse response to antiangiogenic therapies [151]. The weekly administration for 4 weeks of c-Met inhibitor capmatinib (INC280) in combination with bevacizumab (10 or 15 mg/kg iv) was tested in 2017 in a phase Ib trial in young and adult patients with glioblastoma multiforme, progressed after one or two previous lines, in order to demonstrate the maximum tolerated dose of INC280 [151]. However, it is still not used in common clinical practice.

## 2. Discussion

Many achievements have been made in our understanding of clinical importance and biological mechanisms of c-MET/HGF pathway. However, many questions still reclaim an answer to find the definitive way to implement this detailed knowledge and change the clinical practice. Aberrant HGF/MET pathway plays as an oncogenic driver, since dysregulation of c-MET and HGF has been implicated in cancer pathogenesis as it is involved in the mechanisms of cell proliferation and survival, invasion, and metastasis. Increased c-MET expression has been observed in various solid cancers and it is responsible for resistance in most of them. Strategies targeting this pathway, such as monoclonal antibodies (mAbs), have been shown only to achieve stable disease but not to prolong survival in advanced cancers [152–154]. Rational design of novel agents targeting both HGF dependent and HGF-independent MET activation, careful selection of patients for clinical trials, and development of biomarkers are the key for future success in targeting MET pathway. Although the c-MET hyperexpression is certainly associated with a worse prognosis, the use of c-MET inhibitors is not conclusive in all cases presenting c-MET hyperexpression. Firstly, it is mandatory to standardize the detection of c-MET/HGF expression levels in clinical practice and to identify the cut-off level in order to state the high expression. Secondly, MET inhibitors tested in different trials did not give us the expected results. Lastly, it is essential to reveal the main points of connections between c-MET/HGF and other pathways in order to clarify mechanisms of resistance and develop more efficient drugs against them.

## Data Availability

No data were used to support this study apart from those cited within the text as references.
